# The Diverse Roles of Phagocytes During Bacterial and Fungal Infections and Sterile Inflammation: Lessons From Zebrafish

**DOI:** 10.3389/fimmu.2020.01094

**Published:** 2020-06-05

**Authors:** Tanja Linnerz, Christopher J. Hall

**Affiliations:** Department of Molecular Medicine and Pathology, Faculty of Medical and Health Sciences, University of Auckland, Auckland, New Zealand

**Keywords:** zebrafish, phagocytes, macrophages, neutrophils, infection, sterile inflammation, innate immunity

## Abstract

The immediate and natural reaction to both infectious challenges and sterile insults (wounds, tissue trauma or crystal deposition) is an acute inflammatory response. This inflammatory response is mediated by activation of the innate immune system largely comprising professional phagocytes (neutrophils and macrophages). Zebrafish (*danio rerio*) larvae possess many advantages as a model organism, including their genetic tractability and highly conserved innate immune system. Exploiting these attributes and the live imaging potential of optically transparent zebrafish larvae has greatly contributed to our understanding of how neutrophils and macrophages orchestrate the initiation and resolution phases of inflammatory responses. Numerous bacterial and fungal infection models have been successfully established using zebrafish as an animal model and studies investigating neutrophil and macrophage behavior to sterile insults have also provided unique insights. In this review we highlight how examining the larval zebrafish response to specific bacterial and fungal pathogens has uncovered cellular and molecular mechanisms behind a variety of phagocyte responses, from those that protect the host to those that are detrimental. We also describe how modeling sterile inflammation in larval zebrafish has provided an opportunity to dissect signaling pathways that control the recruitment, and fate, of phagocytes at inflammatory sites. Finally, we briefly discuss some current limitations, and opportunities to improve, the zebrafish model system for studying phagocyte biology.

## Introduction

The zebrafish (*danio rerio*) is a well-established model organism used to study a variety of biological and pathological processes. These studies range from developmental biology, genetics (1), cancer (2, 3), neurobiological diseases/neurodegeneration (4), cardiovascular diseases (5), to metabolic (6) and infectious diseases (7–9). Zebrafish embryos and larvae offer unique properties, as they are externally fertilized, thus allowing easy access to the developing embryo throughout its rapid life cycle. Moreover, adult zebrafish can generate a large number of offspring on a weekly basis, and the larvae are optically transparent, a physical trait that can be exploited using transgenic reporter lines for non-invasive live imaging. With respect to live imaging immune responses, transgenic lines that label different types of phagocytes (Table 1), enable the observation of the inflammatory response to injuries and infections in a living animal. Furthermore, the concurrent use of fluorescently-labeled pathogens in infection studies allows the real-time observation of host-pathogen interactions *in vivo*. Besides the imaging potential and genetic amenability, the zebrafish model offers the capacity for high-throughput drug screening to facilitate antimicrobial discovery and in-depth studies of virulence factors.

**Table 1:** Examples of transgenic lines routinely used to visualize phagocytes in larval zebrafish.

	**Transgenic line(s)**	**Experimental use**	**References**
Myeloid-progenitors	*Tg(spi1:EGFP)^*pA*301^*	Cell labeling (whole cell)	(10)
	*Tg(zpu.1:EGFP)^*df*5^*		(11)
Neutrophils	*Tg(mpx:EGFP)^*i*114^*	Cell labeling (whole cell)	(12)
	*Tg(zMPO:GFP)*		(13)
	*Tg(mpx:Dendra2)*	Cell tracking (photoconversion)	(14)
	*Tg(mpx:Gal4)^*i*222^;UAS-E1b:Kaede)^*s*1999*t*^*		(15)
	*Tg(lyz:GAL4.VP16)^*i*252^; (UAS-E1b:Kaede)^*s*1999*t*^*		(16)
	*Tg(mpx:EGFPCAAX)^*gl*27^*	Cell labeling (cell membrane)	(17)
	*Tg(lyz:EGFP)^*nz*115^; Tg(lyz:DsRED2)^*nz*50^*	Cell labeling (whole cell)	(18)
Macrophages	*Tg(mpeg1:EGFP)^*gl*22^*	Cell labeling (whole cell)	(19)
	*Tg(mpeg1:mCherry)^*gl*23^*		
	*Tg(mpeg1: mCherryCAAX)^*sh*378^*	Cell labeling (cell membrane)	(20)
	*Tg(mpeg1: mCherry-F)^*ump*2^*	Cell labeling (cell membrane)	(21)
	*Tg(mpeg1:Dendra2)*	Cell tracking (photoconversion)	(14)
	*Tg(csf1ra:GFP)^*sh*377^*	Cell labeling (whole cell)	(22)
	*Tg(mpeg1:tdTomato-CAAX)^*xt*3^*	Cell labeling (cell membrane)	(23)
	*Tg(mfap4:mTurquoise2)^*xt*27^*	Cell labeling (whole cell)	(24)
	*Tg(mfap4:tdTomato)^*xt*12^*		
	*Tg(mfap4:tdTomato-CAAX)^*xt*6^*	Cell labeling (cell membrane)	
	*Tg(mfap4:dLanYFP:CAAX)^*xt*11^*		
Eosinophils	*Tg(gata2^*high*^:eGFP)*	Cell labeling (whole cell)	(25)

Another major advantage lies in the fact that the human and zebrafish genomes share high homology and the immune system is highly conserved. Even though the developmental origin of the zebrafish immune system differs to some extent from their mammalian counterparts, all major relevant immune cell types have been described in the fish including phagocytic myeloid cells of the innate immune system (26, 27). The innate immune system provides the first line of defense against invading pathogens and is comprised of physical barriers, biochemical effector molecules such as complement factors, antimicrobial peptides, cytokines (chemokines, interferons, and interleukins) and phagocytes. A major effector function of the complement system is to opsonize pathogens and to recruit professional phagocytic cells, such as macrophages and neutrophils (28).

Macrophages and neutrophils are highly migratory cells, which are both capable of phagocytosis and subsequent killing of pathogens. Phagocytosis plays a central role in the defense against invading pathogens and in tissue inflammation and the successive process of healing, where macrophages and neutrophils remove cell debris and restore tissue homeostasis (29, 30). Besides the recognition of opsonins, phagocytosis can also be triggered by the direct binding of pathogen-associated molecular patterns (PAMPs) to pattern recognition receptors (PRRs) on macrophages and neutrophils (29, 30). Once pathogens are internalized, they reside in an intracellular vacuole, the phagosome, which further matures to the phagolysosome where effective killing mechanisms are initiated (31). Additionally, membrane-bound or intracellular-residing Toll-like receptors (TLRs), which belong to the group of PRRs, contribute to the effective recognition of pathogens and the activation of phagocytes (32). The activation of TLRs activates downstream signaling pathways such as nuclear factor kappa-light-chain-enhancer of activated B cells NF-κB, which results in the production and release of pro-inflammatory cytokines by professional phagocytes (33). Transgenic zebrafish reporter lines have been generated utilizing NF-κB recognition sequences and promoters of immune-response genes (including pro-inflammatory cytokines) enabling the differentiation of neutrophil and macrophage activation states (Table 2). These lines have been instrumental in beginning to reveal that the functional heterogeneity of larval zebrafish phagocytes is similar to that of their mammalian counterparts (42).

**Table 2:** Examples of transgenic lines routinely used to visualize and differentiate phagocyte activation states.

**Activation marker**	**Transgenic line(s)**	**Expression confirmed in**	**References**
*il1b* expression	*TgBAC(il1b:egfp)^*sh*445^*	Neutrophils and macrophages	(34)
	*Tg(il1b:EGFP-F)^*ump*3^*	Neutrophils and macrophages	(35)
	*TgBAC(il1b:NTR-EGFP)^*tyt*205^*	Defined as myeloid cells^*^	(36)
*irg1* expression	*Tg(irg1:EGFP)^*nz*26^*	Macrophages only	(37)
*nfkb* expression	*Tg(Nf-kB:EGFP)^*nc*1^*	Macrophages	(38, 39)
	*Tg(8xHs.NFκB:GFP,Luciferase)^*hdb*5^*	Defined as immune cells^*^	(40)
*tnfa* expression	*TgBAC(tnfa:GFP)^*pd*1028^*	Macrophages	(41)
	*Tg(tnfa:eGFP-F)*^*ump*5^	Macrophages	(42)

* *Yet to be confirmed as neutrophils or macrophages*.

The roles of neutrophils and macrophages are often complementary to each other during inflammatory responses, however, the kinetics of their recruitment can be variable, depending on the source of the insult. Neutrophils are usually the first responders after tissue injury and invasion of pathogens, except if patrolling tissue-resident macrophages encounter the microbes first (43). Regardless of the source of the insult, the second professional phagocyte population is commonly recruited shortly thereafter. Both phagocytes react to tissue damage and infection primarily by phagocytosis of foreign particles or tissue debris. Whereas, neutrophils have a higher microbicidal activity through degranulation, the production of reactive oxygen species (ROS), and are capable of neutrophil extracellular trap formation (44, 45); macrophages destroy pathogens and debris intracellularly in the phagolysosome for antigen-presentation, and additionally release cytotoxic factors and initiate chemokine and cytokine production (31, 46). All of the described pathways and the downstream components related to the innate immune response against pathogens or injuries are remarkably conserved between zebrafish and mammals. One interesting property of the zebrafish larval immune system is that the adaptive arm of the immune system takes ~3–4 weeks to develop (47). This creates the exclusive opportunity to study the innate response without interference of adaptive immunity in the early embryonic and larval stages. Furthermore, pharmacologic and genetic techniques exist to specifically deplete phagocyte subsets in larval zebrafish, including the use of liposomal clodronate for macrophage ablation (21) and transgenic lines for nitroreductase-mediated ablation of neutrophils or macrophages (Table 3). Using these ablation techniques, the specific contribution of neutrophils and macrophages to inflammatory responses can be dissected.

**Table 3:** Examples of transgenic lines routinely used to manipulate phagocyte numbers.

**Cell type ablated**	**Transgenic line(s)**	**References**
Macrophages	*Tg(mpeg1:Gal4FF^*gl*25^;UAS-E1b:nfsB-mCherry^*c*264^)*	(19, 48)
	*Tg(cfms:Gal4.VP16)^*il*86^;UAS:nfsB. mCherry)^*i*149^*	(49)
Neutrophils	*Tg(lyz:ntr-p2A-LanYFP)^*xt*14^*	(23)
	*Tg(-8.mpx:KalTA4 ^*gl*28^; UAS-E1b:nfsB-mCherry)^*c*264^*	(50)

Over the last 20 years, the zebrafish has evolved as a model organism for many infectious diseases, including bacterial [reviewed in Neely (7)], fungal [reviewed in Rosowski et al., (9)], viral [reviewed in Varela et al., (8)] and parasitic infections (51). Here we focus on studies examining the phagocyte responses to specific bacterial and fungal infections that have revealed fundamental insights into a spectrum of phagocyte responses, from those that are host protective to those that are detrimental. We also discuss how modeling sterile inflammation in larval zebrafish has enabled a deeper understanding of the signaling systems that regulate the directed movement of phagocytes druing inflammation.

## Phagocyte Responses During Bacterial and Fungal Infection

**Figure 1:**
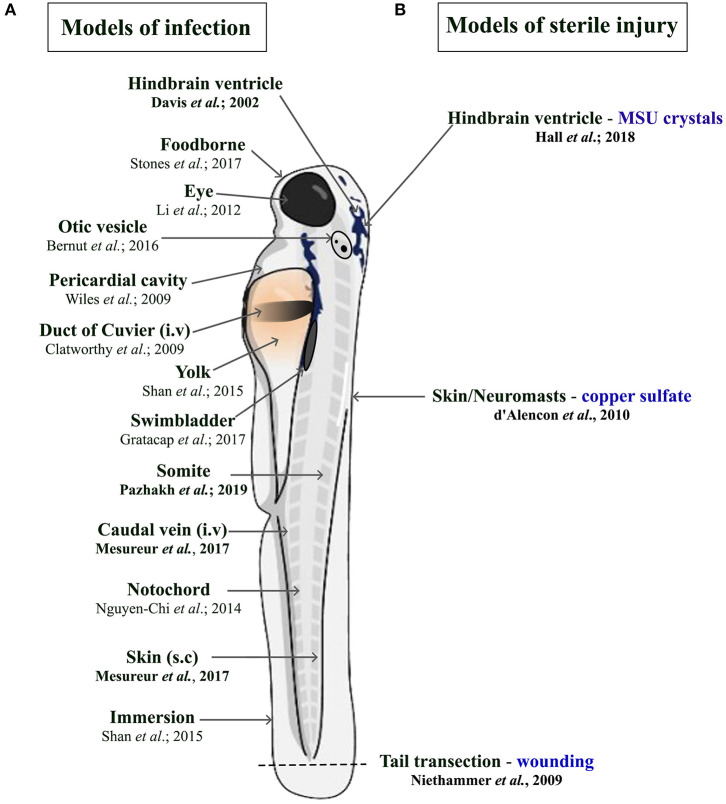
Schematic illustration of the different delivery routes in larval zebrafish for pathogens. (**A**, left side) and to model sterile inflammation (**B**, right side). In the sterile injury section, the stimulus is specified by blue writing. All delivery routes show an exemplary reference, with the ones covered in this review highlighted in bold.

There is a constant need to study infectious diseases and develop novel treatment strategies, especially in the context of growing antibiotic resistance, nosocomial infections, superinfections, and (re-)emerging new pathogens. In many cases, patients rely on a proper innate immune response as a first line of defense, particularly immunocompromised patients. This qualifies the zebrafish as a suitable model due to temporal segregation in the development of innate and adaptive immunity. In addition, infectious challenges can be readily delivered to different anatomical sites within larval zebrafish depending on the microorganism being used and the particular innate immune cell response under investigation (Figure 1A). Many significant studies have utilized the zebrafish model to further our understanding of the host response to important viral [reviewed in Varela et al., (8)] and parasitic infections (51). For the purpose of this review, we have chosen to focus on examples of bacterial and fungal infections that illustrate the heterogenous nature of phagocyte responses. These include host protective phagocyte functions and those that are detrimental, such as facilitating the dissemination of infection or promoting tissue damage.

### Bacterial Infections

Modeling bacterial infections in zebrafish has contributed significantly to our understanding of the early innate immune response toward numerous bacterial infections in humans [reviewed in Neely (7)]. Professional phagocytes play an essential role in limiting bacterial growth and eradicating infection. However, bacteria have evolved different strategies to delay or avoid efficient killing mechanisms in phagocytes. In the following section, we focus on the zebrafish response to *Mycobacterium marinum* (*M. marinum*), as well as studies using *Mycobacterium leprae* (*M. leprae*), *Burkholderia cenocepacia* (*B. cenocepacia*), and *Staphylococcus aureus* (*S. aureus*) that show different ways in which the phagocyte response can be host protective or harmful.

#### The Macrophage Response to *Mycobacterium marinum* Is Largely Host Protective

A classic example of how phagocytes, particularly macrophages, provide a host protective function has been shown in the tuberculosis-like zebrafish disease model using the closely related natural fish pathogen *M. marinum*. Tuberculosis, which is caused by *Mycobacterium tuberculosis* (*M. tuberculosis*), is a persistent major health threat worldwide and remains astonishingly successful in infecting millions of people every year (52). Long-existing conventional views on human tuberculosis pathogenesis have been challenged in the last decade using the closely related pathogen *M. marinum*, to model a tuberculosis-like disease in zebrafish.

Granulomas are clinical hallmark features of tuberculosis and are highly organized structures consisting of infected macrophages at their cores surrounded by lymphocytes, necrotic cell debris (the caseum) and a fibrotic cell layer (53). The granuloma has been generally viewed as a compact barrier and static structure, which restricts bacterial growth and thereby limits their spread (54, 55). Elegant studies in *M. marinum*-infected larval zebrafish have revealed that early granuloma-like structures can form independent of an adaptive immune response, where mycobacteria are predominantly engulfed by macrophages. This initiates the expansion of the granuloma-like structure through the recruitment of uninfected macrophages (56, 57). The recruitment of macrophages is thereby dependent on the bacterial secreted protein ESAT6 (encoded by the RD1 virulence locus) that drives *matrix metallopeptidase 9* expression in epithelial cells neighboring infected macrophages (58, 59). Infected macrophages then quickly undergo cell death and are engulfed by newly arriving uninfected macrophages resulting in accelerated *M. marinum* proliferation and cellular expansion of the granuloma (60). Tracking of individual macrophage responses has also revealed that some infected macrophages leave the granuloma to disseminate the infection by establishing secondary granulomas (60). A more recent study has also shown that when macrophage supply becomes limiting there is a transition from a granuloma that supports mycobacterial growth within macrophages to one favoring macrophage necrosis and the discharge of mycobacteria into the extracellular granuloma milieu (61).

Studies in zebrafish have also revealed several cues that contribute to the onset of macrophage necrosis, including alterations in levels of the pro-inflammatory cytokine tumor necrosis factor (TNF). Transient knockdown experiments using Morpholinos against the TNF-receptor were able to dissect the pleiotropic role of TNF during *M. marinum* pathogenesis (62). In this study, decreased TNF levels induced a hypo-inflammatory state accompanied by augmented mycobacterial growth and accelerated granuloma formation, which ultimately led to enhanced necrosis of macrophages, granuloma breakdown and extracellular proliferation of *M. marinum* (62). Surprisingly, a similar outcome was achieved when excessive TNF levels were present (63). While co-injected recombinant TNF in *M. marinum*-infected zebrafish larvae initially reduced mycobacterial burden, macrophages underwent necroptosis (programmed necrosis) induced by a RIPK1-RIPK3-dependent mechanism, which in turn enhanced mitochondrial ROS production (64). Exploiting the relative ease of exposing larval zebrafish to chemical inhibitors, it was shown that two pathways cooperate to induce this ROS-dependent macrophage necrosis via an inter-organellar circuit (65). Initially, mitochondrial ROS activates ceramide production via the lysosomal enzyme acid sphingomyelinase (aSM). Ceramide, in turn, activates the cytosolic protein BAX, which promotes calcium flow through ryanodine receptors (RyR) from the endoplasmatic reticulum back into the mitochondria. The influx of calcium overloads the macrophage mitochondria, ultimately leading to the activation of the mitochondrial matrix protein cyclophilin D, which induces necrosis (65). This TNF-mediated necrosis mechanism was shown to be conserved for *M. marinum* and *M. tuberculosis*-infected human macrophages (65). The detailed molecular dissection of these TNF-responsive pathways offers a wide array of potential new druggable targets, which have already been applied and validated in the zebrafish tuberculosis model. Promising novel treatments involve inhibitors for cyclophilin D (such as Alisporivir), aSM-blocking drugs (the tricyclic antidepressant Desipramine), calcium channel-blocking drugs (LTCC inhibitors such as Verapamil), RyR-blockers (Dantrolene), or ROS scavengers (64, 65). Many of those drugs are currently in clinical trials or have been approved for the treatment of other diseases. Besides balancing adequate TNF levels in tuberculosis progression, new potential routes for treatment could furthermore include maintaining stable macrophage numbers (61) and specifically targeting macrophages with drug-loaded nanoparticles or liposomes, such as Rifampicin, in the early course of the disease (66, 67).

**Figure 2:**
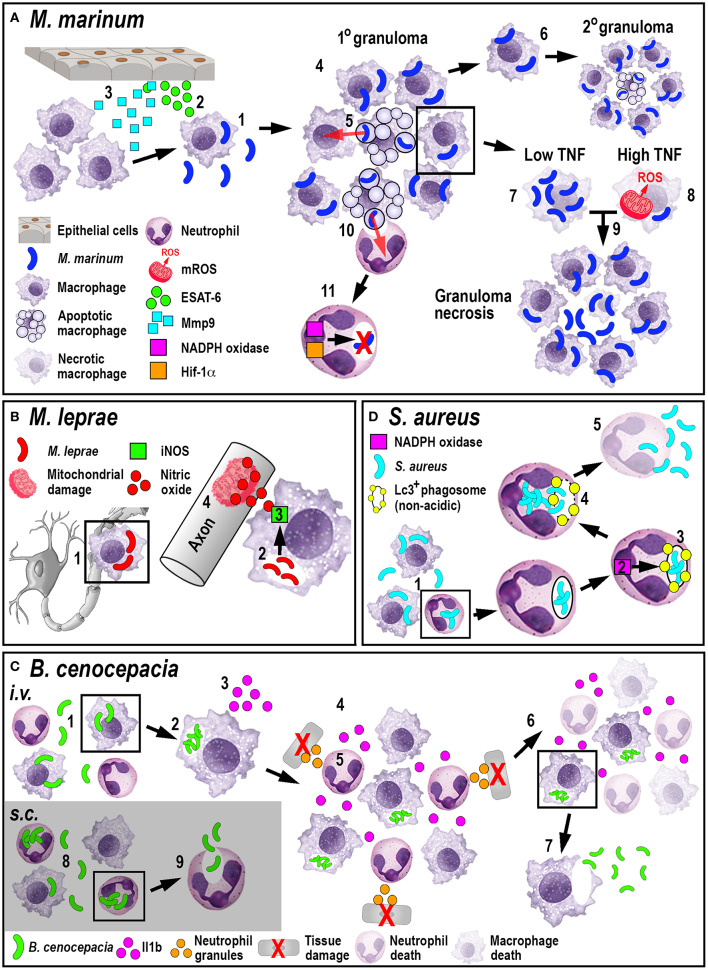
Schematic illustration of the phagocyte responses to the bacterial pathogens *M. marinum* **(A)**, *M. leprae* **(B)**, *B. cenocepacia* **(C)**, and *S. aureus* **(D)**. **(A)** Macrophages phagocytose *M. marinum* (1) and release ESAT-6 (2). ESAT-6-driven Mmp9 production by epithelial cells leads to macrophage recruitment (3) and granuloma formation (4). Newly-arriving macrophages become infected by engulfing dying infected macrophages (5). Infected macrophages can establish secondary granulomas (6). Low TNF levels promote intracellular bacterial growth and macrophage necrosis (7). High TNF levels promote mROS production within infected macrophages that, although initially bactericidal, also leads to necrosis (8). Necrosis results in the release of bacteria into the extracellular milieu (9). Neutrophils can phagocytose infected macrophage debris (10) and kill *M. marinum* by NADPH oxidase-mediated ROS production and Hif-1α-dependent reactive nitrogen species production (11). **(B)** *M. leprae*-infected macrophages migrate along nerve axons (1), where PGL-1 (2) stimulates iNOS-driven nitric oxide production in macrophages (3) that damages mitochondria in adjacent axons (4). **(C)** Following *i.v*. delivery, macrophages phagocytose *B. cenocepacia* (1) providing a replication niche (2). Infected macrophages produce Il1b (3) that attracts neutrophils and macrophages (4), leading to tissue damage resulting from degranulating neutrophils (5). The inflammatory response also leads to myeloid cell ablation that favors the survival of infected macrophages (6). *B. cenocepacia* can disseminate through non-lytic escape from infected macrophages (7). Following *s.c*. infection, neutrophils phagocytose *B. cenocepacia* (8) but are inefficient in killing the bacteria and instead release the bacteria into the extracellular milieu (9). **(D)** Following phagocytosis of *S. aureus* by neutrophils (1), NADPH oxidase activity (2) contributes to the formation of non-acidic Lc3-positive phagosomes (3) that provide a replication niche. Phagosome membrane damage results in the release of bacteria into the cytosol (4), neutrophil death and bacterial dissemination (5).

The role of neutrophils in tuberculosis infection is less clear. While mammalian *in vivo* studies investigating the role of neutrophils during tuberculosis are conflicting (68, 69), zebrafish studies have shown that neutrophils appear to be less important in controlling infection. Although mycobacteria can evade direct phagocytosis by larval zebrafish neutrophils, caspase-mediated cell death of infected macrophages within the granuloma attracts neutrophils, which phagocytose dying macrophages. After this indirect uptake of mycobacteria, neutrophils can directly kill the bacteria by NADPH oxidase-mediated ROS production (70). Even though neutrophils do not appear to be essential to control tuberculosis infection, higher bacterial burdens in later stages of infection have been shown to be accompanied by neutropenia (71). Moreover, forced production of reactive nitrogen species in neutrophils through manipulation of hypoxia-inducible factor 1 (Hif-1α) signaling prior to mycobacteria infection, can induce protection in the host (72). Manipulating either mycobacterial neutrophil evasion strategies or the HIF-1 pathway offer interesting new routes for potential therapeutic interventions. Collectively, these studies investigating the larval zebrafish innate immune response to *M. marinum* have greatly enhanced our understanding of *M. tuberculosis* pathogenesis (Figure 2A) and uncovered new mechanistic insights that may allow for the development of promising new treatments.

#### The Macrophage Response to *Mycobacterium leprae* and *Burkholderia cenocepacia* Damages the Host

A close relative to *M. marinum* and *M. tuberculosis* is *M. leprae*, a non-motile bacterium that causes leprosy in humans. This bacterium grows at 30°C, which makes it difficult to study in mammalian animal models adequately. However, it renders the poikilothermic zebrafish an excellent model organism, which develops clinical symptoms comparable to the human disease (73). A distinct feature of *M. leprae* infection is a widespread demyelinating neuropathy, which manifests as a disorganization and decompaction of myelin sheaths and subsequent axonal damage (74). As with other Mycobacteria species, initial infection and replication occurs in macrophages. Surprisingly, the neurological disease is not directly caused by the pathogen *per se*, but by patrolling infected macrophages. This was demonstrated using a combination of confocal and transmission electron microscopy techniques in larval zebrafish (73). Macrophages were shown to interact with an *M. leprae*-specific component of the outer cell membrane, a triglycosylated phenolic glycolipid 1 (PGL-1), which led to inducible nitric oxide synthase (iNOS)-driven production of neurotoxic nitric oxide. This macrophage source of reactive nitrogen species then caused mitochondrial damage in adjacent axons (Figure 2B). There are currently two hypotheses for how infected macrophages can reach the nerves: one possibility is through an overlying skin lesion that allows for direct seeding of macrophages from a granuloma into a nearby peripheral nerve (75). The second suggests that infected macrophages, which are not enclosed in a granulomatous structure, extravasate from the blood vessels, to patrol axons, similar to their behavior under homeostatic conditions (73, 76). The latter hypothesis would support how leprosy manifests as such a widespread neuropathy in comparison to other mycobacterial diseases.

Similar to *M. leprae* infection, the macrophage response to another opportunistic pathogen *B. cenocepacia* can be detrimental to the host. *B. cenocepacia* belongs to the *Burkholderia cepacia complex* (*Bcc*) and can emerge as an opportunistic pathogen, particularly in cystic fibrosis patients and immunocompromised individuals (77). *B. cenocepacia* is extremely virulent in the zebrafish model, and macrophages were shown to be vital for initial infection and replication (78). Depending on the infection route, intravenously (*i.v*.) or subcutaneously (*s.c*.), live imaging experiments within infected larvae revealed that phagocytes engaged with *B. cenocepacia* in different ways. If *B. cenocepacia* was administered *i.v*., neutrophils and macrophages were both recruited to the infection site. However, only few bacteria were phagocytosed by neutrophils triggering degranulation that resulted in tissue damage and increased bacterial burden. Following *s.c*. infection, neutrophils predominantly phagocytosed *B. cenocepacia* but failed to kill the bacteria. Instead infected neutrophils adopted a circular morphology and ejected the bacteria back into the extracellular space, suggesting non-lytic exocytosis or a NET-based mechanism that was unable to destroy *B. cenocepacia* effectively (79).

In contrast, macrophages predominantly phagocytosed bacteria following *i.v*. delivery and engaged with *B. cenocepacia* at later stages following *s.c*. infection. Regardless of the administration route, *B. cenocepacia* failed to efficiently replicate within macrophage-depleted hosts, resulting in enhanced survival (79). This effect on survival was partially dependent on macrophage-derived Il1b, which induced both host-protective and fatal pro-inflammatory consequences. Additionally, expression analysis showed a global downregulation of the macrophage- and neutrophil-marking genes *mpeg1* and *mpx* after infection, suggesting systemic myeloid cell death through massive inflammation, bestowing a survival advantage specifically to infected macrophages through an unknown mechanism. After intracellular replication in macrophages, *B. cenocepacia* was shown to utilize a non-lytic escape mechanism to infect neighboring cells after leaving the macrophage vacuole, which resulted in a systemic and fatal infection (78). *B. cenocepacia* infection provides an excellent example of how phagocytes demonstrate diverse responses during infections (Figure 2C), where macrophages exacerbate disease outcome instead of protecting the host.

#### Neutrophils Provide an Intraphagocytic Niche for *Staphylococcus aureus*

*S. aureus* is a gram-positive opportunistic pathogen, usually residing on the skin and in nasal cavities of healthy carriers. In immunocompromised individuals, especially in hospitals, *S. aureus* is one of the leading causes of fatal bacteremia/sepsis, skin infections, pneumonia, endocarditis, and osteomyelitis (80). Another serious complication is the growing emergence of antibiotic-resistant strains, such as the methicillin-resistant (MRSA), and vancomycin-resistant (VRSA) *S. aureus* strains, which additionally complicate effective and life-saving treatments. The control of systemic *S. aureus* infection in larval zebrafish was strongly dependent on phagocytes, as neutrophil and macrophage ablation caused exponential growth of GFP-tagged *S. aureus* in the circulation and rapid death of infected larvae (81). Chemical ablation of either neutrophils or macrophages revealed that both cell populations were indispensable for controlling infection, however, macrophages seemed to be more important for this process as they predominantly phagocytosed the bacteria (82). Furthermore, *S. aureus* uses neutrophils as an intraphagocytic niche for host immune evasion (82, 83). Once *S. aureus* is phagocytosed by neutrophils, the bacteria utilize the host autophagy machinery to successfully evade intracellular killing mechanisms. After internalization, the neutrophil-intrinsic NADPH oxidase prompts Lc3-associated phagosome formation, which contains the bacteria and provides a protective niche as these phagosomes do not acidify. *S. aureus* subsequently damage the Lc3-associated phagosomal membrane, resulting in bacterial proliferation and dissemination (Figure 2D) (83).

This host immune evasion strategy is particularly important in the context of mixed-strain infections, where a drug-resistant mutant can be present in a bacteria population. It had been demonstrated in previous experiments that the injection of an equal ratio of two differentially labeled *S. aureus* strains, resulted in one of the injected strains dominating the infected larvae, despite identical initial proliferation (82). This preferential expansion was the result of a few individual bacteria that exclusively survived within neutrophils (82, 83). Furthermore, this behavior correlates with antibiotic resistance. It has been demonstrated that if multiple *S. aureus* strains were present in a host with differential antibiotic resistances (drug-resistant vs. drug-sensitive), the drug-resistant strain predominated even if only sub-curative amounts of the antibiotic were present (84). This effect was not observed in phagocyte-depleted larvae, suggesting that drug-resistant *S. aureus* strains are more likely to use the autophagy-mediated immune evasion strategy. Understanding the diverse mechanisms through which phagocytes engage with pathogens and how different bacteria can avoid or exploit host innate immune responses, promises to reveal new anti-microbial strategies that are of clinical need in the age of increasing antibiotic resistance.

### Fungal Infections

Modeling fungal infections in zebrafish has gained increasing attention within the last decade. Two significant reasons have contributed to this popularity. Firstly, many fungal pathogens prefer lower incubation temperatures (33°C), which resemble the lungs, outer limbs and skin temperature of humans (85), which is easily practicable with zebrafish embryos. Secondly, zebrafish embryos and larvae resemble late-stage HIV-infected patients, as they do not possess a functional adaptive immune system, rendering this model ideal to study opportunistic fungi (86). Comprehensive reviews describing the modeling of different fungal infections in larval zebrafish have previously been covered elsewhere (9, 87, 88). Here we highlight studies exploring the host response to three specific fungal pathogens (*Aspergillus fumigatus* (*A. fumigatus*), *Talaromyces marneffei* (*T. marneffei*), and *Cryptococcus neoformans* (*C. neoformans*) that encompass a range of phagocyte responses, from providing protective niches against host immunity to mediating inter-phagocyte fungal transfer and fungal dissemination.

#### Macrophages Can Protect Fungal Pathogens From Neutrophil-Mediated Killing

Spores of *A. fumigatus*, an environmental fungus, are inhaled on a daily basis but can cause invasive or pulmonary aspergillosis in immunocompromised individuals (89). Once the dormant spores (conidia) are taken up by a host, a developmental switch to filamentous, invasive hyphae occurs, a process called germination (89). Fungal germination is considered a key event in pathogenesis because neutrophils respond to the hyphal form with the initiation of highly efficient killing mechanisms intracellularly (ROS/RNS and Mpx-dependent) and extracellularly, such as NETosis (90–92). At this point, *A. fumigatus* infection appears contradictory: while hyphae are a necessary virulence factor, germination ultimately entails fungal clearance due to the activation of neutrophils. The role of macrophages in disease progression is less clear. While *in vitro* studies support an important role in killing conidia (93), mouse *in vivo* studies have shown contradicting results (94, 95).

**Figure 3:**
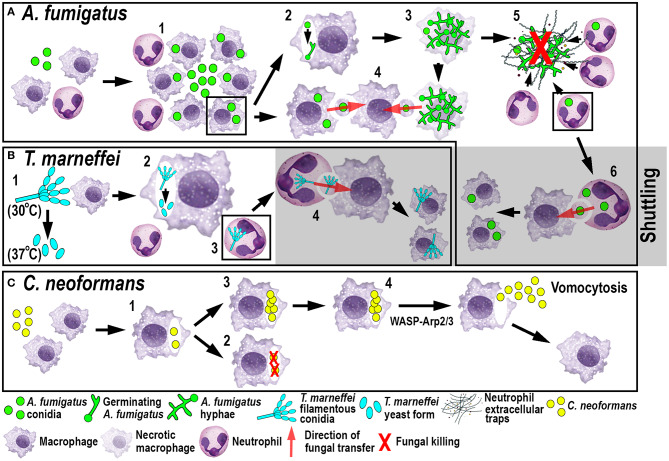
Schematic illustration of phagocyte responses to the fungal pathogens *A. fumigatus* **(A)***, T. marneffei* **(B)** and *C. neoformans* **(C)** in larval zebrafish. **(A)** Macrophages phagocytose *A. fumigatus* conidia and form tight clusters around the fungus, which inhibits fungal germination (1). Fungal germination can occur in the late phagosomes of infected macrophages (2) causing macrophage necroptosis (3). Transfer of *A. fumigatus* conidia can occur from infected and dying macrophages to recipient macrophages (4). Neutrophils kill *A. fumigatus* hyphae with their effector functions, including phagocytosis and NETosis (5). Infected neutrophils can transfer *A. fumigatus* to recipient macrophages through shuttling (6). **(B)** *T. marneffei* exists as filamentous conidia at 30°C and as pathogenic yeast form at 37°C (1). *T. marneffei* spores can transition to the yeast form within macrophages (2). Neutrophils can also phagocytose *T. marneffei* conidia (3) and transfer them to recipient macrophages through shuttling (4). **(C)** Macrophages phagocytose *C. neoformans* (1) where it can be killed (2) or persist and proliferate (3). WASP-Arp2/3 regulates vomocytosis and fungal dissemination (4).

A recent elegant larval zebrafish study was able to shed light on the role of macrophages in *A. fumigatus* infection (Figure 3A) (9). The study demonstrated that following infection, macrophages phagocytosed injected conidia (91) and formed tight clusters around the fungus (9). The observed macrophage-driven phagocyte clusters resembled “fungal” granulomas (aspergillomas) (96), which is commonly observed in other infections, such as *M. tuberculosis/M. marinum*. This recent study has revealed that the macrophage clustering creates a protective niche for the spores by inhibiting the switch to fungal germination by an unknown mechanism (9). This delay promotes the persistence of the fungus by preventing neutrophil recruitment and subsequent neutrophil-mediated killing (9). Moreover, the retardation of fungal germination allows certain fungicidal drugs, such as voriconazole, to target and kill predominantly *A. fumigatus* hyphae (97). In light of growing antifungal resistances and inexplicable treatment failures, the larval zebrafish *A. fumigatus* infection model provides an ideal platform for studying drug efficacy and their mechanistic impact on aspergillosis (97). Once the infection progressed further, fungal germination occasionally occured in the late phagosome causing subsequent macrophage necroptosis (98). In some instances, lateral cell-cell transfer from dying to naïve-recipient macrophages was observed using high-resolution confocal microscopy of fluorescently-labeled macrophages and *A. fumigatus*, a process called metaforosis, which further restricted germination of the fungus (99). Elucidating the dichotomous role of macrophages in creating a protective niche for *A. fumigatus*, while simultaneously promoting control of germination (9, 99), may create new avenues for therapeutic strategies.

Another fungal pathogen that uses macrophages as a protective niche to prevent neutrophil-mediated killing is *T. marneffei* (Figure 3B). *T. marneffei* (formerly classified as Penicillium) infects predominantly HIV and AIDS patients in southeast Asia and can result in a lethal systemic infection (talaromycosis) (100). This fungus primarily infects macrophages, which has also been demonstrated in the zebrafish infection model using different routes of infection. Even though neutrophils interacted and phagocytosed conidia, the spores were preferentially taken up by macrophages (17). *T. marneffei* is thermally dimorphic, which means it exists as filamentous conidia at moderate temperatures (in the environment) and switches to a more pathogenic yeast form at 37°C (inside the host). Interestingly, once macrophages phagocytosed the conidia in the ectothermic zebrafish model, the transition to the yeast morphology occurred regardless of the lower incubation temperature of the host. This suggests that alternative cues can supersede the requirement for a specific temperature, such as the intracellular milieu of macrophages (17). This might also partially explain why macrophages are the preferred location for the initial infection and the proliferation of *T. marneffei* within the host. As with *A. fumigatus, T. marneffei* used macrophages as a protective niche to escape neutrophil-mediated killing, which is primarily achieved through the myeloperoxidase activity abundantly found in their granules.

#### Shuttling-a New Mechanism of Fungal Transfer Between Phagocytes

Recently, a novel mechanism of pathogen transfer between phagocytes (shuttling) has been identified in zebrafish studies using *A. fumigatus* and *T. marneffei* infections (Figures 3A,B) (101), which may be of therapeutic relevance in potential treatments. This transfer of pathogens has been shown to be unidirectional and exclusively from neutrophils to macrophages, while both phagocyte populations remain alive and intact, at the time of the exchange and afterwards. Thus, far, shuttling was only observed within the first 4 h following infection in which single or multiple conidia were transferred to naïve or pre-loaded macrophages. This pathogen exchange mechanism happened through a direct cell-to-cell contact between donor neutrophil and recipient macrophage, which was demonstrated using sophisticated live imaging techniques with differentially labeled phagocyte populations. Phagocytosed conidia resided in a membrane-lined subcellular compartment within the neutrophil and were entirely transferred to the macrophage, suggesting not only pathogen but also phagosome exchange between the phagocytes. This shuttling mechanism was initiated by β-glucan, an integral component of the fungal cell wall. This newly discovered pathogen exchange mechanism was also conserved in isolated mouse neutrophils and macrophages (101). Macrophages recognized β-glucan and participated in shuttling partially through Dectin-1 signaling, which could only be demonstrated *in vitro*, as the zebrafish ortholog of this receptor has not yet been identified. At present, it is unclear if this phenomenon presents a host-defense strategy or a fungal escape mechanism to avoid the unfavorable neutrophil intracellular compartment and access the preferred macrophage niche (9, 17).

#### Dissemination of Infection by Vomocytosis

The fungus *C. neoformans*, which is also able to persist and proliferate in macrophages, uses a different phagocyte escape mechanism. *C. neoformans* is an environmentally occurring fungus and can cause life-threatening meningitis in immunocompromised patients (102). Even though the phenomenon of non-lytic exocytosis has already been observed previously following mouse and human *in vitro* studies (103), it has only recently been directly visualized for the first time in macrophages using a larval zebrafish *C. neoformans* infection model (20). This phagosome expulsion or “vomocytosis” maintains the pathogen, as well as the phagocyte, alive and intact. Moreover, in the case of *C. neoformans*, macrophages do not serve as a protective niche but limit the dissemination of the fungus within the host. Thus, vomocytosis from macrophages into the extracellular space helps to promote fungal growth (Figure 3C). Moreover, *in vitro* mammalian studies showed that cryptococci-loaded phagosomes formed dynamic actin structures dependent on Wiscott-Aldrich-Syndrome-protein/actin-related protein 2/3 (WASP-Arp2/3) signaling, which were able to counteract non-lytic expulsion and could thereby provide a route for novel pharmacological intervention (103). This non-lytic escape mechanism does not appear to be limited to macrophages and has also recently been described in neutrophils *in vitro* (104). Interestingly, this resembles the observations described earlier in neutrophilic *Bcc* infections (79) suggesting that zebrafish may be a suitable model to investigate further this mechanism of exocytosis, as well as potential interventions.

## Phagocyte Responses During Sterile Inflammation

In addition to being vital for the host response to microbial challenges, a cellular innate immune response is also essential for tissue and wound repair. Similar to that observed during infection, the host response to such “sterile” insults is dominated by the recruitment of phagocytic cells, in particular neutrophils and macrophages. The zebrafish offers several established models of sterile inflammation, from acute injury and chemical insults to crystal injections (Figure 1B) (105–108). The following section describes studies that have utilized zebrafish models of sterile inflammation and uncovered mechanistic insights into how neutrophil numbers are controlled during sterile inflammation and how macrophages help orchestrate neutrophil migration.

### Wound-Induced Inflammation

Tissue damage usually triggers a local inflammatory response, which is considered sterile as the host reaction originates from a non-pathogen insult. The recruitment of immune cells, in particular phagocytes, is crucial when physical barriers are compromised to eliminate infiltrating pathogens and clear cellular debris during the process of tissue healing. The timely resolution of this inflammatory response is critical for the restoration of normal tissue function and to avoid prolonged tissue damage. In many inflammatory conditions such as COPD, asthma, rheumatoid arthritis, osteoporosis and atherosclerosis (109), the resolution process is disturbed, which results in a chronic and often incurable manifestation of the disease. The classical wounding model in the zebrafish comprises of tail fin transections or incisions in larvae between 2 and 4 days of development, whereas relatively few studies have induced wounds by needle stabbing at more anterior sites (110). Paired with the use of phagocyte-labeled transgenic zebrafish lines, this model enables the visualization of immune cell behaviors during the initiation and the resolution phase of sterile inflammation. In the initiation phase, neutrophils are typically the first responders to wounding and actively migrate toward the wound following a chemotactic gradient.

#### An Early Role for Hydrogen Peroxide in Attracting Neutrophils

**Figure 4:**
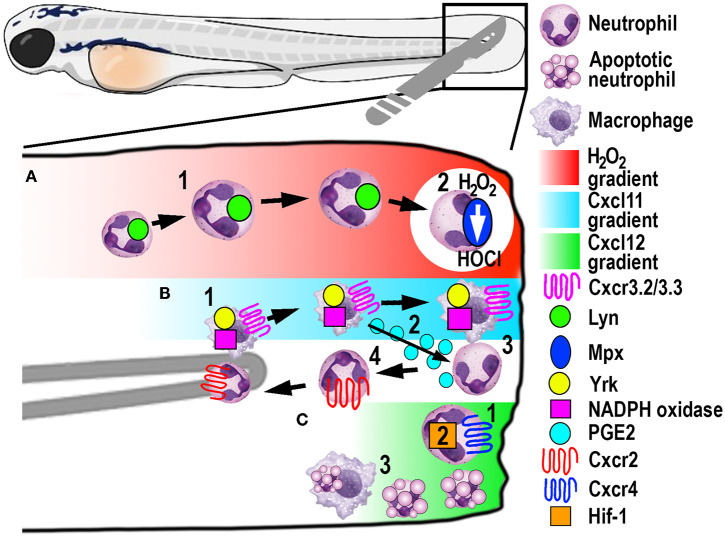
Schematic illustration of the signaling pathways and mechanisms that help control phagocyte migration and abundance during larval zebrafish acute tail fin injury. **(A)** A gradient of H_2_O_2_, generated at the wound margin, is sensed by neutrophils through oxidation of Lyn, leading to directed neutrophil migration (1). Neutrophil-delivered Mpx consumes H_2_O_2_ producing hypochlorous acid (HOCl) (2). **(B)** Macrophage arrival at the wound site is promoted by NADPH oxidase activity and Yrk, in addition to Cxcr3.2/Cxcl11(1). In this context, Cxcr3.3 acts as a scavenger receptor to negatively regulate Cxcr3.2 function. Macrophage-delivered PGE2 promotes neutrophil retrograde chemotaxis (2) together with NADPH/Yrk-dependent contact-mediated guidance from macrophages (3). Cxcr2 signaling also contributes to initiate neutrophil retrograde chemotaxis (4). **(C)** Cxcr4/Cxcl12 signaling contributes to the retention of neutrophils at the wound site (1) along with Hif-1 activation (2). Macrophages at the wound site also remove apoptotic neutrophil debris (3).

A significant contribution made to neutrophil biology by exploiting the zebrafish model was uncovering a role for hydrogen peroxide (H_2_O_2_) as a chemotactic signal for the earliest arriving neutrophils (105). Combining fluorescently-labeled neutrophil transgenic lines with the ability to visually measure H_2_O_2_ concentrations in real-time using the ratiometric sensor HyPer, a non-myeloid derived H_2_O_2_ gradient was discovered, for the first time, in and around wounds *in vivo* (105). This elegant study revealed that peak H_2_O_2_ production, generated via the dual oxidase (Duox) enzyme, was strongest at the wound margin and was necessary for recruitment of the earliest arriving neutrophils. Soon after this discovery, another study employing the zebrafish model revealed that neutrophils sensed the local gradient of H_2_O_2_ through activation of the Src family kinase (SFK) Lyn by oxidation of the cysteine residue C466 (111). Of significance, these findings were confirmed in mouse and human *in vitro* experiments (111). A further study focussed on understanding the transient nature of the H_2_O_2_ chemotactic signal. The study discovered that following arrival at the wound, neutrophils immediately begin to reduce the wound-derived H_2_O_2_ through the intrinsic myeloperoxidase (Mpx) enzyme that catalyzes an H_2_O_2_-consuming reaction to produce halides (112). This H_2_O_2_-driven recruitment of neutrophils (Figure 4A) has been validated in several *in vitro* and *in vivo* models, ranging from invertebrates to humans (111, 113, 114).

#### Neutrophil Retrograde Chemotaxis Helps Resolve Neutrophilic Inflammation

Once neutrophils assist with clearing DAMPs, cell debris and invading pathogens, they need to be removed in a timely fashion from the wound site to limit collateral tissue damage. The dominant theory for several decades suggested that macrophages phagocytose and eliminate apoptotic neutrophils, conferring a central role for macrophages in resolving sterile inflammation (115). Through live imaging, a zebrafish study discovered that an alternative mechanism of neutrophil migration away from the wound site (retrograde chemotaxis) also contributed to the resolution of inflammation (13). Many studies have since followed to further elucidate different mechanisms for the initiation of neutrophil retrograde migration from wound sites to the vasculature. The conserved occurrence of this mechanism has since been shown in human neutrophils (116) and mouse models (117), and appears complementary to regulated neutrophil apoptosis during inflammation resolution.

Macrophages play a major role in controlling neutrophil retrograde migration. Although they arrive slightly later than neutrophils at sites of tissue injury, macrophages also sense a chemotactic gradient through the closely related SFK Yes-related kinase Yrk (14). Once macrophages arrived at the wound, they elicited a contact-mediated guidance program toward neutrophils, which was dependent on Yrk and p22phox, an integral component of the NADPH oxidase complex (Figure 4B) (14). These findings demonstrated a central role for ROS-activated SFK signaling cascades that involved multiple SFK family members, especially Lyn and Yrk, in the phagocyte recruitment and resolution phase of sterile inflammation, respectively (14, 111). Despite the discovery of this contact-mediated mechanism, one of the main roles of macrophages in wound healing, in addition to the clearance of cell debris, is phagocytosis of apoptotic neutrophils (Figure 4C). A recent study investigated in more detail the role of macrophages in restoring normal tissue homeostasis (118). Live imaging in larvae depleted of macrophages revealed the accumulation of neutrophil apoptotic bodies at the wound site, which resulted in the persistence of inflammation (118). Moreover, the same study showed the most abundant eicosanoid Prostaglandin E2 (PGE2) was produced by macrophages and was essential for the subsequent promotion of retrograde migration (Figure 4B), highlighting the interplay of both mechanisms for efficient resolution of inflammation (118).

Additionally, chemotaxis plays a superordinate role during inflammation resolution, as genetic depletion of chemokines or their receptors abrogated retrograde migration despite the presence of macrophages (107). Even though Cxcr2 was originally implicated in neutrophil migration toward infection, this chemokine receptor showed additional functions in the initiation of retrograde migration in tissue injury (107, 119). The chemokine receptor-ligand pair Cxcl8a (interleukin-8)/Cxcr2 proved to be crucial to orchestrate the initiation of neutrophil migration away from the wound (Figure 4B) (107, 119), which has also been shown in human neutrophils (107). Remarkably, another chemokine receptor-ligand pair, Cxcl12/Cxcr4, had the opposite role in this process, as activation of this signaling axis resulted in retention of neutrophils at the wound site (Figure 4C) (120). This work is particularly interesting in the context of chronic inflammatory diseases because samples from patients with rheumatoid arthritis or chronic inflammatory lung diseases have been shown to have increased CXCR4 expression on infiltrating neutrophils (121). Hence altered chemokine profiles and receptor expression could play crucial roles in establishing chronic diseases by retaining neutrophils at sites of sterile inflammation and inhibiting retrograde migration.

Recently, a novel chemokine axis has been implicated in the recruitment of macrophages to wounds. The Cxcr3/Cxcl11 receptor-ligand pair was shown to contribute to macrophage migration toward tail wounds using knockouts of the respective receptors in zebrafish (122). The CXCR3 receptor exists as three paralogs in zebrafish (Cxcr3.1, Cxcr3.2, and Cxcr3.3), whereby Cxcr3.2 and Cxcr3.3 antagonistically function during macrophage recruitment. As both receptors share the same ligand, Cxcr3.2 promotes macrophage migration toward the wound, whereas Cxcr3.3 negatively regulates Cxcr3.2 function by acting as a scavenger receptor for Cxcl11 (Figure 4B) (122).

In another zebrafish study, the involvement of hypoxia added a further signaling axis to those that regulate retrograde migration (16). Mimicking hypoxic conditions pharmacologically or genetically by manipulating Hif-1α, neutrophil apoptosis at the injury site was decreased, as was the rate of retrograde chemotaxis (Figure 4C) (16). Thus, hypoxia/HIF-1 activation delays the resolution of the inflammation after tissue injury and offers a pharmacologically amenable target for potential therapeutic interventions. Because this resolution mechanism includes multiple possibilities for druggable targets, it is noteworthy that neutrophils that underwent retrograde migration did not show a primed inflammatory state or have obvious functional differences to those during steady-state (123). These findings suggest that pharmacological induction of reverse migration may be an attractive new strategy for therapeutic treatments, especially in the context of chronic inflammatory conditions where excessive neutrophil recruitment and retention contribute to tissue destruction.

### Chemical and Crystal-Induced Inflammation

One of the major drawbacks of the beforementioned studies involving tail fin transections is the necessity to manipulate every larva individually. This not only exacerbates, and in some instances, precludes high-throughput approaches but can additionally introduce experimental variations within, as well as between, experiments. By immersing larval zebrafish in copper sulfate, a local inflammatory response can be generated in a high-throughput fashion through inducing neuromast cell death (106). Neuromasts are mechanoreceptors that are dispersed superficially along the whole body surface and belong to the lateral line system of fish and amphibia, which senses water pressure and direction. The rapidly induced apoptosis of neuromasts lead to a local inflammatory response characterized by neutrophil and macrophage recruitment, similar to tail fin transection. This experimental setup can be coupled to a (semi-)automatic quantitative readout with transgenic lines possessing fluorescently labeled phagocytic cells to accelerate and facilitate analysis. Using this approach, a vast number of small molecules can be screened for anti-inflammatory properties at multiple steps during the inflammatory response, from the initiation to the resolution phase, depending on the time of drug administration (106).

A further example of sterile inflammation are crystallopathies, which can be caused by the inhalation of airborne micro- or nanoparticles, the endogenous self-aggregation of misfolded proteins or the supersaturation and subsequent deposition of crystals (124). During excretion of organic metabolites, serum urate levels increase, which can lead to hyperuricemia and the formation of monosodium urate (MSU) crystals. The MSU crystals accumulate in and around joints, which leads to the development of the chronic inflammatory disease, gouty arthritis (125). The MSU crystal deposition causes an acute inflammatory reaction, termed a gout flare, which is extremely painful and usually self-resolves within 1–2 weeks (126). Our group has recently developed a larval zebrafish model of gout to provide new insights into how phagocytes become activated in response to crystal-induced sterile inflammation (108). In this novel zebrafish model, MSU crystals, the causative agent in the development of gouty flares, were locally injected into the hindbrain ventricle of zebrafish larvae. The MSU crystals caused an immediate activation of tissue-resident macrophages and a subsequent inflammatory reaction, mimicking the acute gout flare in the joints of gout patients. The acute inflammatory reaction in the human condition is often connected to increased consumption of alcohol and purine-rich foods, which results in the substantial release of fatty acids (FAs) into the circulation (127). By live imaging metabolic processes [such as mitochondrial ROS (mROS) production; (128)] within macrophages during MSU-driven crystal inflammation, we were able to demonstrate that β-oxidation of FAs fueled macrophage activation through elevated mROS production (108). Moreover, this immunometabolic mechanism was conserved in human macrophages. Through performing a drug repositioning screen to identify drugs that inhibit this immunometabolic mechanism of macrophage activation, we uncovered two drugs (chrysin and piperlongumine) that effectively inhibited inflammation in an *in vivo* mouse model of acute gouty inflammation.

## Concluding Remarks and Future Perspectives

In this review, we have discussed several examples of modeling infectious and sterile inflammation within larval zebrafish and how these models have been utilized to provide novel mechanistic insights into diverse phagocyte functions. These studies have revealed that the examination of phagocyte-pathogen interactions and microbial evasion strategies are greatly facilitated by the live imaging potential of transparent zebrafish larvae and the ever-expanding number of transgenic reporter lines. This direct observation potential has helped to understand how pathogens can use specific immune cells as intracellular niches or shields, in particular macrophages, and avoid phagolysosomal killing mechanisms (9, 17, 56, 57, 59, 78, 101). Furthermore, specific dissemination or escape strategies have now been successfully demonstrated or validated using zebrafish infection studies, when it was often not possible to observe or dissect such mechanisms previously in an intact animal setting. One such mechanism is non-lytic exocytosis (vomocytosis), which leaves the pathogen as well as the phagocytic cell intact and has only been observed in cell culture studies before (20, 78). Another important example is the discovery of a dissemination mechanism of fungal conidia in a process termed shuttling, where spores are leaving the unfavorable neutrophil environment and are transferred to their preferred macrophage niche (101).

In addition to live imaging, taking advantage of the genetic tractability of the zebrafish system has allowed for the examination of pathogen virulence factors and the discovery of host determinants of susceptibility or resistance toward infections. Several genetic screens for pathogenic elements (129, 130) or relevant host genes (131) have been successfully performed in zebrafish. The latter study discovered the leukotriene A4 hydrolase (LTA4H) locus as a susceptibility determinant for *M. marinum* and *M. tuberculosis* infections in zebrafish and humans, respectively. In several successive studies, LTA4H has been shown to be ultimately responsible for TNF levels by catalyzing the final reaction in lipid mediator leukotriene B4 (LTB4) synthesis, which could either promote or inhibit TNF production in zebrafish or human tuberculosis (63–65). Analogous to the detrimental effect of imbalanced TNF levels, LTA4H deficiency or excess both resulted in hypersusceptibility toward *M. marinum* and *M. tuberculosis*, as well as increased macrophage necrosis. Importantly, the discovery of this essential genetic host factor using the zebrafish led to the discovery of a single nucleotide polymorphism in the human LTA4H promoter that was associated with phagocyte recruitment, survival and response to anti-inflammatory treatment in patients with tuberculous meningitis.

Not only is the zebrafish model a valuable tool for the initial discovery of novel mechanisms, but it also provides an excellent platform to perform chemical screens to identify drugs that actively target those pathways for therapeutic benefit. As an example, a compound screen searching for small molecules that influence neutrophil retrograde migration and apoptosis identified a drug derived from a Chinese medicinal herb, tanshinone IIA, that was able to accelerate inflammation resolution. The drug was able to simultaneously induce neutrophil apoptosis and promoted retrograde migration in larval zebrafish, an activity that was conserved when examining human neutrophils (15).

Despite the multiple advantages of the zebrafish, there are currently still certain limitations present in this animal model. For one, there is still a prominent lack of available antibodies, which not only hampers advances in zebrafish proteomics but also impedes the discovery and differentiation of phagocyte subsets. Antibody-based staining and selection techniques are routinely used in rodent and human studies, which is currently not possible to the same extent using the zebrafish model. Moreover, the lack of knowledge regarding the degree of functional heterogeneity in immune cell lineages, in particular macrophages and neutrophils, precludes certain in-depth studies on the same level as it is currently possible in mammalian models. However, the zebrafish system is offsetting these limitations with the more recent development of transgenic lines that mark activated phagocytes, which will aid in identifying distinct phagocyte subpopulations. Additionally, rapid advances in single cell RNA-sequencing technology will help to resolve the uncertainty about the functional heterogeneity of larval macrophage and neutrophil subsets and how they compare to their mammalian counterparts. With the recent and advanced CRISPR/Cas9 technology, host-pathogen interactions and tissue inflammation mechanisms can now be studied in-depth on a molecular and genetic level using cutting-edge genomic engineering techniques. Moreover, the CRISPR/Cas9 technology enables zebrafish researchers to recreate human risk alleles for inflammatory diseases using homology-directed repair mechanisms, which may assist in unraveling how such risk alleles contribute to disease.

## Author Contributions

TL and CH jointly wrote this manuscript.

## Conflict of Interest

The authors declare that the research was conducted in the absence of any commercial or financial relationships that could be construed as a potential conflict of interest.
